# Importance of Cx43 for Right Ventricular Function

**DOI:** 10.3390/ijms22030987

**Published:** 2021-01-20

**Authors:** Kerstin Boengler, Susanne Rohrbach, Norbert Weissmann, Rainer Schulz

**Affiliations:** Institute of Physiology, Justus-Liebig University, 35392 Giessen, Germany; susanne.rohrbach@physiologie.med.uni-giessen.de (S.R.); norbert.weissmann@innere.med.uni-giessen.de (N.W.); rainer.schulz@physiologie.med.uni-giessen.de (R.S.)

**Keywords:** connexin, right ventricle, pulmonary hypertension, tetralogy of fallot, mitochondria

## Abstract

In the heart, connexins form gap junctions, hemichannels, and are also present within mitochondria, with connexin 43 (Cx43) being the most prominent connexin in the ventricles. Whereas the role of Cx43 is well established for the healthy and diseased left ventricle, less is known about the importance of Cx43 for the development of right ventricular (RV) dysfunction. The present article focusses on the importance of Cx43 for the developing heart. Furthermore, we discuss the expression and localization of Cx43 in the diseased RV, i.e., in the tetralogy of Fallot and in pulmonary hypertension, in which the RV is affected, and RV hypertrophy and failure occur. We will also introduce other Cx molecules that are expressed in RV and surrounding tissues and have been reported to be involved in RV pathophysiology. Finally, we highlight therapeutic strategies aiming to improve RV function in pulmonary hypertension that are associated with alterations of Cx43 expression and function.

## 1. Structure of Connexin Proteins and Expression in the Heart

Connexins are a family of transmembrane proteins, which are labelled according to their molecular weight. The human family comprises 21 members with molecular weights between 26 and 60 kDa. Of the connexin protein family, connexin 43 (Cx43) has a broad expression spectrum in cells and organs and is the most studied isoform. Therefore, the present review focusses mainly on the role of Cx43.

The connexin proteins share a similar structure, i.e., an aminoterminus located in the cytosol, two extracellular loops, one intracellular loop and four transmembrane domains. The carboxyterminus, which varies in length between the connexin proteins, is also present in the cytosol. The major role of connexins is the formation of gap junctions, which facilitate the exchange of factors weighing up to 1.5 kDa and thereby mediate electrical and metabolic cell–cell coupling. Such gap junctions are formed by two opposing connexons, each from one cell. The connexon—or hemichannel—itself is composed of six connexin proteins. Whereas the unpaired hemichannels in the plasma membrane remain closed under physiological conditions [1], pathological situations favor their opening, a process leading to dissipation of ionic gradients across the plasma membrane and finally to cell death [2,3]. In addition, Cx43-formed hemichannels may also open upon activation of ryanodine receptors [4]. Cell–cell communication is not only mediated by gap junctions, but is also achieved via extracellular vesicles, in which Cx43 has been detected [5]. Myocardial ischemia/reperfusion injury impairs the secretion of Cx43 into cardiomyocyte-derived extracellular vesicles [6]. In addition to the plasma membrane, Cx43 is localized at the inner membrane of mitochondria isolated from ventricular myocardium, specifically in subsarcolemmal mitochondria (SSM) [7,8]. Cx43, which is encoded in the nucleus, is imported into the SSM in an Hsp90 (heat shock protein 90)/TOM (translocase of the outer membrane)-dependent pathway [9]. Only limited amounts of Cx43 are detected within interfibrillar mitochondria (IFM), which differ from the subsarcolemmal mitochondria in terms of respiration and calcium handling [10,11]. The mechanism responsible for the higher amount of Cx43 in SSM than in IFM is yet unclear.

Several aspects of mitochondrial function are influenced by Cx43, e.g., respiration [12], formation of reactive oxygen species (ROS) [13,14], opening of the mitochondrial permeability transition pore [15] and potassium handling [16]. An aminoterminally truncated Cx43-isoform (Gja1-20k, size 20 kDa) generated by alternative translation is important for the transport of the full-length protein to the plasma membrane and the normal electrical function of the heart [17,18]. Moreover, Gja1-20k is also present in myocardial SSM but not in IFM [19]. The overexpression of Gja1-20k decreases angiotensin II-induced hypertrophy, enhances mitochondrial oxygen consumption and lowers ROS formation [20]. In contrast, the truncated Cx43 isoform Gja1-11k (size 11 kDa) preferentially localizes to the nucleus and limits the progression of the cell cycle [21]. Therefore, the function of Cx43 is not restricted to its classical role as a gap junction protein.

The carboxyterminus of Cx43 contains several amino acids, which can be phosphorylated by different kinases, among them protein kinase C (PKC), mitogen-activated MAP kinase (MAPK), casein kinase 1 or protein kinase B (AKT) (for review see [22]). The phosphorylation of Cx43 regulates the size of gap junctions as well as cell–cell communication. Moreover, phosphorylation of Cx43 seems to be involved in the release of exosomes [23]. In addition, mitochondrial Cx43 is phosphorylated [24]; however, the exact contribution of phosphorylation towards the function of mitochondrial Cx43 is not yet resolved. Gap junctional intercellular communication is also regulated by connexin ubiquitination [25] and by protein–protein interactions [26].

Connexin proteins have been detected in a variety of different organs and cells. In the heart, Cx43 is the most abundantly expressed isoform and is localized in ventricular and atrial cardiomyocytes, but also in endothelial cells, smooth muscle cells and fibroblasts. Cx40 is predominantly expressed in atrial cardiomyocytes, in the cardiac conduction system and is also found in endothelial cells. Endothelial cells also express Cx37.

Table 1 summarizes the localization of Cx37, Cx40 and Cx43 in cardiac compartments or cell types.

The comparison of the amount of Cx43 in the right ventricle (RV) and left ventricle (LV) shows no difference in mouse [34,35], rat [36,37], or human myocardium [29], and similar protein levels are also described for right and left human atria [29].

**Figure 1:**
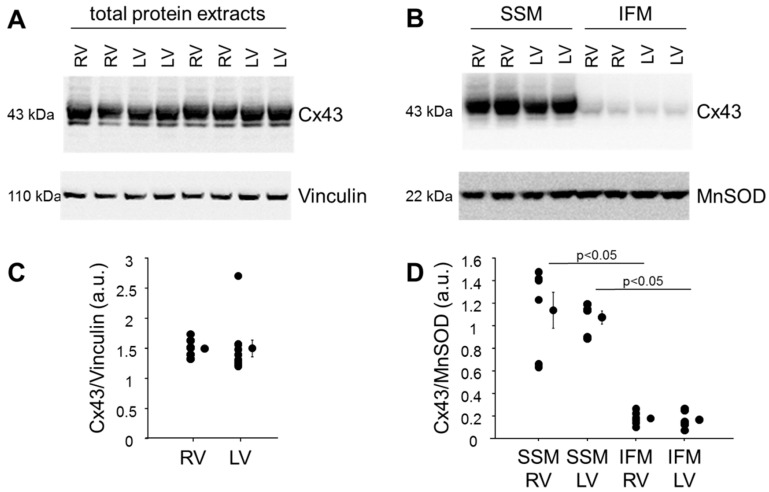
Amount of Cx43 in left and right ventricular total and mitochondrial protein extracts. Western blot analysis was performed for Cx43, Vinculin (housekeeping protein used for total protein extracts) and MnSOD (manganese superoxide dismutase—housekeeping protein used for mitochondrial protein extracts) on proteins isolated from right and left mouse (C57Bl6/J) ventricular tissue (**A**) or on subsarcolemmal (SSM) and interfibrillar (IFM) mitochondrial proteins, isolated from mouse right (RV) and left (LV) ventricular tissue (**B**). A and B demonstrate uncut, representative parts of the Western Blot analysis. The amounts of Cx43 were normalized to Vinculin (**C**, n = 10) or MnSOD (**D**, n = 6). The purity of the mitochondrial preparations was demonstrated by the absence of marker proteins for other cellular compartments (plasma membrane, cytosol and nucleus, Supplementary Materials Figure S1). Data were compared by unpaired Student’s t-test (total proteins) or by two-way ANOVA (mitochondrial proteins). The study was approved by the animal welfare office of the Justus-Liebig University, Giessen, Germany (522_M, approved 29 November 2019).

When analyzing the amount of Cx43 in total protein extracts from right and left mouse ventricular tissue and also in SSM isolated from mouse RV and LV tissue (Figure 1), comparable amounts of Cx43 in total RV and LV protein extracts were detected. Only marginal Cx43 amounts were found in IFM confirming previous data from our and other groups [8,38,39]. These limited amounts of Cx43 within IFM were similar between IFM isolated from RV and LV myocardium.

In contrast, Cx40 is more abundantly detected in right than in left human atria, whereas similar transcript and protein levels are found in human right and left ventricles [29,40].

In summary, Cx43 is the most studied member of the connexin family. Whereas the classical role of Cx43 is the formation of gap junctions and hemichannels at the plasma membrane, recent studies demonstrate a broader spectrum of Cx43 localizations, i.e., within exosomes and mitochondria, and also truncated Cx43 isoforms reveal specific functions. In the heart, Cx43 is evenly distributed within ventricles, atria and ventricular mitochondria.

## 2. Role of Cx43 for the Developing Heart

The contribution of Cx43 towards the development of the heart is studied in different strains of knockout mice as well as in Cx43-overexpressing mice. Homozygous Cx43-deficient mice, in which nearly the whole coding sequence (including the sequences encoding the transmembrane domains) is replaced by the neo^r^ gene (neomycin resistance gene), die shortly after birth from failing pulmonary gas exchange [41]. Despite the fact that Cx43 is expressed in several different organs, only the heart shows abnormal morphologies starting at embryonic day ten with abnormal looping [42]. The analysis of the homozygous Cx43-deficient mice reveals that the conus region overlying the RV outflow tract is enlarged, and double pouches are observed at the base of the pulmonary outflow tract [43]. The RV outflow tract is filled with intraventricular septa and thus the RV outflow tract is blocked. In addition, mice with a Cx43/β-galactosidase fusion protein with dominant negative effects on cell–cell communication reveal malformations of the RV outflow tract and outflow tract obstruction [44]. Abnormal RV outflow tract bulging, trabecular in-growth, and extravasation of contrast material into the RV wall are shown in inducible Cx43-knockout mice using magnetic resonance imaging [45]. The analysis of the expression of Cx43 in rabbit hearts demonstrates a heterogeneous expression of the protein in the RV outflow tract and shows that cells are connected at their lateral sides [46]. Despite the high expression level of Cx43 in the left ventricle, no morphological alterations are described in this heart chamber; however, patterning defects of the coronary arteries are also observed [47]. Mice constitutively overexpressing Cx43 are generally viable and fertile; however, postnatal viability is reduced [48]. Interestingly, mice overexpressing Cx43 exhibit a similar phenotype as Cx43-knockout mice. Here, also an enlargement of the conotruncal region of the RV and narrowing of the pulmonary outflow region is observed [48]. The effects of Cx43 on heart morphology seem to be mediated by cardiac neural crest cells and in the non-crest neural tube [49].

The analysis of hearts from Cx40-knockout mice shows a 33% incidence of double-outlet right ventricle/tetralogy of Fallot (TOF) [50] and also the occurrence of a septum primum defect and ventricular septal defects [51]. Therefore, as well as Cx43, Cx40 seems to play a role in cardiogenesis, especially in the septation process (for review see [52].

Taken together, despite a high expression of Cx43 in the left heart, data from Cx43-deficient, as well as Cx43-overexpressing, mice emphasize the role of Cx43 in the RV outflow tract development. The reason for a similar phenotype in both Cx43-deficient and Cx43-overexpressing mice is unclear at present. Since the expression of Cx40 is unaffected by deletion of Cx43 [41,42], a compensation for the loss of Cx43 function by Cx40 is unlikely; however, mice in which Cx43 is replaced by Cx40 are rescued from postnatal lethality [53] indicating that the two proteins share at least some important functions.

## 3. Connexins in the Diseased Right Ventricle

### 3.1. Tetralogy of Fallot

TOF is the most common cyanotic congenital heart disease and is characterized by ventricular septal defect, obstruction of the right ventricular outflow tract, right ventricular hypertrophy and an over-riding aorta. Whereas the pathomechanisms of TOF are not completely understood up to now, a contribution of Cx43 is suggested. Increased amounts of Cx43 mRNA and protein are detected in the RV outflow tract of children with TOF compared to healthy controls [54], while others describe lower amounts of Cx43 protein in children with TOF compared to those with ventricular septal defect [55]. Data on the distribution of Cx43 at the plasma membrane showed either an enhanced heterogeneous localization of Cx43 in TOF patients [54] or no differences in the amount of Cx43 at the polar compared to the lateral sides of the cardiomyocytes—and also no alterations of Cx43 phosphorylation—between TOF patients and patients with other cardiac malformations [56]. Histone H3 containing the acetylated lysine 18 (H3K18ac) is shown to directly bind to the Cx43 promoter, and the acetylation of H3K18ac is reduced in patients with TOF, which may alter the transcription and subsequently the protein amount of Cx43 [57]. Whereas a point mutation within the Cx43 promoter increases the susceptibility to TOF [57], others found no causal role of single nucleotide polymorphisms within the Cx43 gene for the development of TOF [56] and consider Cx43 mutations not as important contributors in the development of outflow tract abnormalities [58].

In accordance with the phenotype of Cx40 mice, mutations in the *GJA5* gene encoding Cx40 are considered a susceptibility gene for TOF [59].

Patients with corrected TOF have a higher risk of developing arrhythmias and dying due to sudden cardiac death. In a porcine model of repaired TOF, the remodeling of RV conduction and the repolarization properties are associated with a decreased Cx43 protein expression, as well as enhanced lateralization of the protein [60]. A trend towards increased Cx43 lateralization after repaired TOF is also observed in the piglet LV [61]. In addition, a reduced amount of the Cx43 mRNA occurs one year after TOF repair in dogs, although the Cx40 mRNA level is not altered [62].

Table 2 summarizes studies addressing the role of connexins in TOF and repaired TOF.

Taken together, whereas a contribution of both Cx40 and Cx43 towards the development of TOF is suggested, the exact mechanisms by which the proteins are involved in the disease remain elusive.

### 3.2. Pulmonary Hypertension: Connexins in Vascular Cells

Exposure to hypoxia leads to pulmonary vasoconstriction and, consequently, an increase in pulmonary arterial pressure occurs, which is characteristic for pulmonary hypertension (PH). The increase in pulmonary arterial pressure is sustained due to subsequent remodeling of the pulmonary vasculature. Pulmonary arterial smooth muscle cells show enhanced proliferation and decreased apoptosis.

In order to study molecular mechanisms regulating the development of PH, several animal models are studied. The most widely used model is the rat monocrotaline-model, in which upon a single injection of monocrotaline, a pyrrolizidine alkaloid, the animals develop PH with severe remodeling of the pulmonary vasculature and RV hypertrophy approximately within four weeks [63]. This model represents severe PH as it occurs in idiopathic pulmonary arterial hypertension (IPAH) in men. The chronic hypoxia model is also frequently studied and results in PH. In contrast to the monocrotaline model, where the intima of the vessels is also largely affected, chronic hypoxia leads to less severe PH with vessel media thickening and thus represents so-called group 3 PH. Another approach to induce PH uses the vascular endothelial growth factor receptor antagonist Sugen 5416 in combination with chronic hypoxia. In rats, this treatment causes angio-obliterative lesions in the pulmonary arterioles, which resemble the “plexiform” lesions found in patients with IPAH, and thus also induces a severe form of PH [64]. In contrast to the aforementioned methods, banding of the pulmonary artery (PAB) increases RV pressure without affecting and being dependent on the pulmonary vasculature. Animal models of PH are reviewed elsewhere [65,66].

A contribution of connexins towards the development of PH is investigated in vascular cells from animal models as well as in cells isolated from patients with PH. In pulmonary arterial endothelial cells isolated from explanted lungs of PH patients, reduced amounts of both Cx37 and Cx40 are detected [67]. However, others report no differential Cx37 and Cx40 mRNA and protein expression in pulmonary arteries of monocrotaline-treated rats or rats exposed to chronic hypoxia [68]. The use of the phosphodiesterase 5 inhibitor sildenafil, which elevates intracellular amounts of cGMP and supports vasodilation, leads to an upregulation of Cx40 in pulmonary arterial smooth muscle cells from monocrotaline-treated rats and decreases cell proliferation. In the presence of LDN-193189, an antagonist of type II receptor for bone morphogenetic protein (BMPR2), sildenafil fails to stimulate the amount of Cx40 in pulmonary arterial smooth muscle cells from monocrotaline-treated rats, thereby showing the importance of BMPR2 in triggering the sildenafil-induced expression of Cx40 [69].

The expression of Cx43 is studied in different forms of PH and it is shown to decrease in pulmonary arteries isolated from patients with idiopathic PH [70]. Those patients exhibit enhanced levels of micro-RNA1 (miR-1), and the transduction of pulmonary arterial smooth muscle cells with miR-1 induces a downregulation of the Cx43 expression [71]. In contrast to the decreased Cx43 level in patients with IPAH, enhanced amounts of Cx43 are detected in pulmonary arteries derived from patients with chronic hypoxia-induced PH [70]. In pulmonary arteries of rats exposed to chronic hypoxia, the Cx43 mRNA—in contrast to the Cx37 or Cx40 mRNA—is upregulated [68]. An enhanced Cx43 protein amount is also shown in lungs of Sugen 5416/hypoxia-treated rats, an effect dependent on the activation of ASK-1 (apoptosis signal-regulating kinase-1), an upstream regulator of the mitogen-activated protein kinase pathway [72]. A causal role of Cx43 for the development of PH is demonstrated in heterozygous Cx43-deficient mice subjected to chronic hypoxia that were partially protected against PH [70]. Data on connexins in the vasculature in different models of PH are presented in Table 3.

In summary, connexin expression is influenced by PH in vascular cells. Cx40 is downregulated in PH in humans and its stimulation seems to be involved in mediating the protective effects of sildenafil. In contrast, the amount of Cx43 can be either up- or downregulated depending on the exact form of PH and this has to be taken into account when considering Cx43 as a vascular target for the treatment of PH.

### 3.3. Pulmonary Hypertension: Connexins in Right Ventricular Hypertrophy and Failure

PH results from increased pulmonary vascular resistance. Chronic pressure overload stimulates RV hypertrophy, which can compensate for the increased RV afterload by reducing wall stress and energy demand. However, sustained pressure overload induces RV decompensation with dilatation and failure [73]. Since patients with RV decompensation have a higher risk of suffering from arrhythmias, it is worth studying electrophysiological remodeling in animal models of PH. The administration of monocrotaline alters the electrophysical properties of the RV in several aspects. At rapid heart rates, ventricular tachyarrhythmias occur in monocrotaline-treated rats [74]. In addition, a prolongation of action potential duration and increased action potential duration heterogeneity are observed [74]. Whereas in the hypertrophied RV the repolarization is prolonged, the failing RV shows an increase in repolarization heterogeneity [75]. In addition, it is suggested that the atrioventricular node dysfunction observed after monocrotaline treatment is due to changes in the mRNA profiles of ion channels [76]. Monocrotaline not only alters the electrical properties of the RV but also those of the right atrium. Here, monocrotaline induces a substrate for the maintenance of atrial fibrillation due to right atrial re-entrant activity [77].

Since impulse propagation in the heart is mediated via connexin-formed gap junctional channels and the preservation of connexin topology has anti-arrhythmic effects [78,79,80], the alterations of right atrial and ventricular electrophysical properties suggest a deregulation of connexin expression/function in experimental models of PH. The amount of Cx43 is assessed at different time periods after administration of monocrotaline. Compared to control RV samples, decreased amounts of the Cx43 mRNA and protein are detected in RV tissue 4 weeks [81], 5 weeks [82,83] and 6–7 weeks [74] after monocrotaline treatment. The downregulation of Cx43 persists at least until 90 days after monocrotaline administration, when a reduced protein amount of the protein is detected [84,85]. The protein level of Cx43 90 days after injection of monocrotaline is not only reduced in the RV but also in the lung [86], whereas 5 weeks after injection, higher Cx43 protein amounts in the lung are observed [83]. The downregulation of Cx43 in monocrotaline-treated rats is not restricted to the RV; rather, the expression of Cx43 is also altered in other compartments of the heart. In the right atrium, the Cx43 mRNA is reduced 3 weeks after monocrotaline injection [77]. The analysis of the amount of Cx43 in LV tissue of monocrotaline-treated rats revealed non-consistent results: whereas one study demonstrates decreased Cx43 protein levels approximately 4 weeks after administration of monocrotaline [87], others show no alteration of the Cx43 protein 5 weeks after monocrotaline-injection [82]. Data on the phosphorylation of Cx43 in the monocrotaline model are rare. One study indicates decreased phosphorylation of Cx43 4 weeks after monocrotaline-treatment in RV tissue—without analysis of specific phosphorylation sites, whereas the total amount of the protein is not affected [88]. Others describe maintained Cx43 protein amount 4 weeks after monocrotaline administration [89]. Interestingly, despite the maintained Cx43 protein level in monocrotaline-treated rats, the aforementioned studies describe an increased lateralization of Cx43, which is confirmed 5 weeks after monocrotaline injection [90].

Although the monocrotaline model represents a severe model of PH with increased RV overload, direct effect of monocrotaline on the RV cannot be completely ruled out in the above studies. In addition to the monocrotaline model, connexin expression and function are also studied after PAB, where such direct effect of a PH-inducing drug on the RV is absent. In fetal sheep RV tissue, the mRNA of both Cx40 and Cx43 decreases after chronic overload (5 days) [91]. In addition to decreased amounts of Cx40 and Cx43, a decline in the level of the Cx37 mRNA is detected in mouse RV tissue 2 weeks after banding of the pulmonary artery [92]. In papillary muscles isolated 4–6 weeks after the PAB operation, the amount of the Cx43 mRNA and protein declines especially in tissue samples with interstitial fibrosis compared to those without fibrosis. Here, also a disorganization of the intercalated discs is observed [93], making it likely that diminished cell–cell communication contributes to the disturbance of myocyte synchronous activation. In addition, adult sheep subjected to RV pressure overload display enhanced Cx43 lateralization; however, concurrent lateralization of microtubule-associated proteins relevant for Cx43 trafficking suggests that lateralization may represent an adaptive mechanism that prevents uncoupling [94].

Whereas the majority of the studies show a downregulation of Cx43 after PAB, also enhanced amounts of the Cx43 mRNA are detected in RV tissue 7 weeks after the induction of compensated hypertrophy or RV failure by PAB [95]. Data on the electrophysiological properties of the RV in different models of PH, as well as on the expression and localization of Cx43, are given in Table 4.

Taken together, the expression of Cx43 is studied in both the rat monocrotaline and the PAB model. Both experimental approaches demonstrate a downregulation of the Cx43 mRNA and protein, which is paralleled by enhanced lateralization of the protein. Thus, the dysregulation of Cx43 is suggested to contribute to the alterations of action potential propagation and the development of RV hypertrophy.

## 4. Connexins and Treatment of Pulmonary Hypertension

The findings that, in pulmonary hypertension, the expression of connexins, especially Cx43, is reduced and a lateralization of the protein occurs suggest that strategies to treat PH may also affect the amounts of connexins as well as their function. Therefore, different approaches to improve RV function in PH consider the role of connexins and the respective studies are discussed in the following section.

The activation of the vasoconstrictive–proliferative axis of the renin angiotensin system leads to the development of PH, whereas the activation of the counterbalancing axis via the conversion of Angiotensin II to Ang-(1–7) by the angiotensin-converting enzyme 2(ACE2) protects against PH in monocrotaline-treated rats [96] and is a promising strategy in humans [97]. In mice, the infusion of recombinant ACE2 decreases RV hypertrophy and increases RV ejection fraction after PAB. Whereas the expression of Cx37 is downregulated after PAB, the improved RV function after ACE2 infusion is associated with a normalized expression of Cx37 [92]. Possibly, Cx37-mediated cell–cell communication contributes to the protective effects of the ACE2 infusion.

The expression of Cx37 and Cx40 is regulated by the cis-acting factor MEF2 (myocyte enhancer factor 2), the activity of which is reduced in PH. The pharmacological inhibition of class IIa histone deacetylases using MC1568 restores MEF2 activity and increases the levels of the Cx37 and Cx40 mRNAs and, moreover, decreases the proliferation of pulmonary artery endothelial cells isolated from patients with PH and reduces RV systolic pressure and RV hypertrophy in monocrotaline-treated rats [67].

The enhanced proliferation of the pulmonary arterial vascular smooth muscle cells in PH is associated with alterations in the calcium homeostasis. In monocrotaline-treated rats, the expression of the sarcoplasmic calcium ATPase 2a (SERCA2a) is downregulated [74], an effect contributing to electrophysiological remodeling. Accordingly, it is hypothesized that a normalization of the SERCA2a expression will improve vascular remodeling and will reduce pacing-induced ventricular tachyarrhythmia in PH. Indeed, intra-tracheal gene delivery of aerosolized SERCA2a to the lung normalizes the expression level of SERCA2a and of subunits of potassium channels in the RV and accordingly decreases the occurrence of pacing-induced ventricular tachyarrhythmia. In addition, the amounts of the Cx43 mRNA and protein, which are reduced after the administration of monocrotaline, are increased after SERCA2a delivery and nearly reach the levels detected in the control group [74].

A study by Lee et al. [84] focusses on the role of potassium channels in PH. Here, the administration of nicorandil, which has vasodilatory properties due to regulating the opening of ATP-dependent potassium channels and the release of nitric oxide, to monocrotaline-treated rats decreases the RV systolic blood pressure, the weight of the RV and inhibits the proliferation of smooth muscle cells. Compared to the injection of monocrotaline alone, the administration of nicorandil enhances the protein amount of Cx43. A further elevation of the Cx43 expression is observed following the combined administration of nicorandil and colchicine, which prevents microtubule polymerization.

Another therapeutic approach to ameliorate PH in monocrotaline-treated rats is the administration of cilostazol, which exerts antiproliferative effects on vascular endothelial and smooth muscle cells [86]. The use of cilostazol reduces RV systolic blood pressure and RV hypertrophy. This protective effect is associated with the enhanced expression of Cx43 in the RV and in the lung and it is suggested that the preserved protein level of Cx43 in cilostazol-treated animals contributes to the reduced RV wall stress and hypertrophy.

In addition, sildenafil treatment prevents the downregulation of Cx43 in the RV of monocrotaline-treated rats [85].

The endothelin receptor antagonist bosentan, which is capable of improving PH [98] and hemodynamics in PH patients [99], also reduces RV systolic blood pressure, but not the RV/LV thickness ratio in monocrotaline-treated rats [90]. In contrast to the aforementioned studies, the investigation by Tan et al. [90] focuses on the distribution of the Cx43 at the RV cardiomyocyte plasma membrane. Here, a lateralization of Cx43 is observed after the administration of monocrotaline. Such alteration of the Cx43 distribution is not visible after bosentan treatment.

The aforementioned studies all demonstrate that protection against PH is associated with preservation of connexin expression and/or localization. In contrast, monocrotaline-treated rats have an increased number of Cx40- and Cx43-expressing CD4^+^ and CD8^+^ T cells in lung tissue and the administration of the unspecific gap junction blocker carbenoxolone decreases the expression of these connexins and reduces RV hypertrophy in monocrotaline-treated rats [100]. These data show that the inhibition of connexins decrease the monocrotaline-induced pulmonary inflammatory response.

In Table 5, therapeutic approaches to improve RV function in PH, which are associated with alterations of connexin expression and/or function are presented.

Taken together, whereas the amounts of connexin proteins are reduced and the distribution of Cx43 at the plasma membrane is disturbed in PH, different therapeutic approaches to improve RV function in PH are associated with a normalization of connexin expression and its localization at the plasma membrane. However, a causal role of connexins in the reduction in RV systolic blood pressure and RV hypertrophy is yet unproven.

## 5. Conclusions

**Figure 2:**
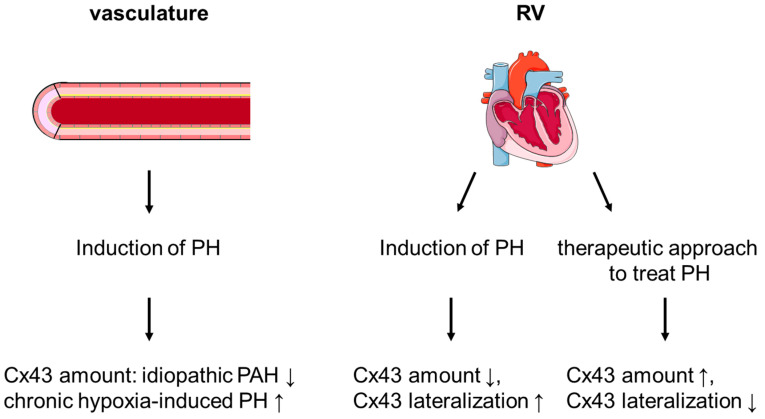
Schematic representation of the expression and localization of Cx43 in pulmonary hypertension (PH). In the vasculature, the amount of Cx43 depends on the exact form of PH. In the right ventricle (RV), PH is associated with a downregulation of Cx43 as well as with enhanced lateralization of the protein. Therapeutic approaches to treat PH can maintain Cx43 expression and localization. Schematic representation of the heart: copyright © 2005, Les Laboratoires Servier. PAH: pulmonary arterial hypertension.

A contribution of connexins, especially Cx43, towards the development of LV hypertrophy and failure is demonstrated in several studies; however, the role of connexins in the diseased RV is less studied. In general, the protein amount of Cx43 is similar in RV and LV tissue, both at the level of the total protein extracts and within mitochondria. In the RV, connexins seem to play a role in the tetralogy of Fallot and in PH. Here, alterations in the expression of connexins are detected in vascular cells as well as in RV tissue. Frequently, Cx43 is downregulated in PH and an increased lateralization of the protein is observed. Whether or not mitochondrial Cx43 contributes to the development of PH is yet unclear. Therapeutic approaches that improve RV function in PH are often associated with a normalization of the Cx43 expression (see Figure 2). Whereas data on the signal transduction and mechanisms leading to Cx43 dysregulation are limited and no causality between dysfunctional Cx43 and the development of RV hypertrophy and failure is proven yet, the available data legitimate the need for further studies on the role of Cx43 in the diseased RV.

## Figures and Tables

**Table 1:** Localization of connexins in the heart.

Connexin	Localization	Reference
Cx37	Vascular endothelial cells, endothelial cells of the endocardium	[27]
Cx40	Vascular endothelial cells	[27]
	Fibroblasts	[28]
	Atria	[27,29]
	Smooth muscle cells	[30]
	His bundle	[31]
	Bundle branches	[32]
	Distal Purkinje system	[33]
Cx43	Atria	[27,29]
	Fibroblasts	[28]
	Smooth muscle cells	[30]
	His bundle	[31]
	Distal Purkinje system	[33]
	Ventricles	[29]

**Table 2:** Connexins in tetralogy of Fallot.

Species	Model	Result	Reference
Human	TOF patients (1.3 ± 0.9 years) and normal controls (1.8 ± 0.9 years)	TOF: Cx43 mRNA ↑	[54]
Human	TOF patients (4.6 ± 0.9 months) Control: patients with ventricular septal defect, without RVOT pathology (3 w—4.5 months)	TOF: Cx43 protein ↓ Cx43 irregularly distributed on the cardiomyocyte surface	[55]
Human	TOF patients (0–2 years, >2 years) Control: age-matched patients with pulmonary atresia with or without ventricular septal defects, double chamber RV or truncus arteriosus communis	TOF: no alteration of Cx43 distribution and phosphorylation; Point mutations within the Cx43 gene no cause for TOF development	[56]
Human	TOF patients (median age: 7.5 months) Normal controls: (median age: 48 months)	TOF: H3K18ac acetylation ↓	[57]
Human	TOF patients (median age: 7.5 months	SNP in the Cx43 promoter is associated with ↓ Cx43 mRNA	[57]
Pig	rTOF or sham surgery, 23 ± 1 w after surgery	rTOF: remodeling of RV conduction and repolarization properties; Cx43 protein ↓in epi- not in endocardium; Cx43 lateralization ↑ endocardium	[60]
Pig	rTOF or sham surgery, 23 ± 1 w after surgery	rTOF: LV tissue: trend towards ↑ Cx43 lateralization, Cx43 protein unchanged	[61]
Dog	rTOF or no surgery, 1 year after surgery	rTOF: RVOT: Cx43 mRNA ↓, Cx40 mRNA unchanged	[62]
Human	300 patients with congenital heart dis ease, including 224 with outflow tract anomalies, 152 with TOF, 50% of the cohort <1 year of age	In patients with congenital heart disease: no amino acid changing mutations in the gene encoding Cx43	[58]

LV: left ventricle; rTOF: repaired tetralogy of Fallot; RV: right ventricle; RVOT: right ventricular outflow tract; TOF: tetralogy of Fallot; ↑: increased; ↓: decreased.

**Table 3:** Pulmonary hypertension: connexins in vascular cells.

Species	Model	Result	Reference
Human	PAECs from PH patients and control donor lungs	PH: Cx37 and Cx40 mRNA ↓	[67]
Rat	Chronic hypoxia for 3 w MCT (60 mg/kg), 4 w, isolation of intra-pulmonary arteries	Chronic hypoxia, MCT: no alteration of Cx37, Cx40 mRNA and protein Chronic hypoxia: Cx43: ↑ mRNA and protein MCT: Cx43 unchanged	[68]
Rat	MCT (60 mg/kg) or vehicle, isolation of PASMC after 28d, treatment of cells with sildenafil or sildenafil + LDN-193189	PH: sildenafil ↑ Cx40 mRNA and protein in PASMC, effect reversed by LDN-193189	[69]
Human	Patients with IPAH or PH due to hypoxemic chronic lung disease	iPH: Cx43 ↓ CH-PH: Cx43 ↑	[70]
Mouse	Cx43^+/+^ and Cx43^+/−^ mice undergoing hypoxia-induced PH	PH: Cx43^+/−^ ↓ pulmonary arterial muscularization, inflammatory infiltration	[70]
Rat	PASMC transduced with miR-1	Cx43 expression ↓	[71]
Rat	Sugen5416 (20 mg/kg) followed by 2 w hypoxia and 3 w normoxia; GS-444217 (10 µM) during normoxia	Sugen 5416/hypoxia: Cx43 protein ↑ in lung tissue, effect reversed by GS-444217	[72]

IPAH: idiopathic pulmonary arterial hypertension; MCT: monocrotaline; PAEC: pulmonary arterial endothelial cells; PASMC: pulmonary arterial smooth muscle cells; PH: pulmonary hypertension; ↑: increased; ↓: decreased.

**Table 4:** Pulmonary hypertension: connexins in RV hypertrophy and failure.

Species	Model	Result	Reference
Rat	MCT (60 mg/kg) or saline, analysis after 6–7 w	PH: action potential duration ↑, action potential duration heterogeneity ↑, Cx43 mRNA and protein ↓	[74]
Rat	MCT (60 mg/kg) or control, analysis after 5–8 w	RVH: prolongation of ventricular repolarization; Failing RV: repolarization heterogeneity ↑	[75]
Rat	MCT (60 mg/kg) or saline, analysis after 4 w	PH: atrioventricular node dysfunction associated with derangement in the ion channel transcriptome	[76]
Rat	MCT (60 mg/kg) or saline, analysis after 3 w	PH: pacing-induced atrial fibrillation ↑, conduction slowing and rotor activity, transcriptome derangement related to hypertrophy, inflammation, fibrosis in RA; RA: Cx43 mRNA ↓	[77]
Rat	MCT (60 mg/kg) or no treatment, analysis after 4 w	PH: Cx43 protein ↓	[81]
Rat	MCT (60 mg/kg) or saline, analysis after 5 w	PH: RV: Cx43 mRNA and protein; LV: Cx43 unchanged ↓	[82]
Rat	MCT (70 mg/kg) or saline, analysis after 5 w	PH: RV: Cx43 protein ↓; lung: Cx43 protein ↑	[83]
Rat	MCT (60 mg/kg) or saline, analysis after 90d	PH: Cx43 protein ↓	[84]
Rat	MCT (65 mg/kg) or saline, analysis after 90d	PH: RV: Cx43 protein ↓; lung: Cx43 protein ↑	[85]
Rat	MCT (75 mg/kg) or saline, analysis after 90d	PH: RV: Cx43 protein ↓; lung: Cx43 protein ↓	[86]
Rat	MCT (60 mg/kg) or saline, analysis after approximately 4 w	PH: RV and LV: Cx43 protein ↓	[87]
Rat	MCT (60 mg/kg) or saline, analysis after 4 w	PH: Cx43 protein lateralization ↑; Cx43 phosphorylation ↓; Cx43 protein unchanged	[88]
Rat	MCT (60 mg/kg) or saline, analysis after 4 w	PH: Cx43 protein lateralization ↑; Cx43 protein unchanged	[89]
Rat	MCT (60 mg/kg) or saline, analysis after 5 w	PH: Cx43 protein lateralization ↑; Cx43 protein unchanged	[90]
Sheep	PAB in fetal sheep for 1 h or 5d; control: unbanded twin fetus	PAB: RV: Cx40 and Cx43 mRNA ↓ 5d	[91]
Mouse	PAB or sham surgery, analysis after 2 w	PAB: RV: Cx37 mRNA ↓	[92]
Rat	PAB or sham surgery, analysis after 4–6 w	Papillary muscles with fibrosis: Cx43 mRNA and protein ↓ compared to tissue without fibrosis; disorganized intercalated discs	[93]
Sheep	Injection of sephadex beads, analysis after 60 d	PH: lateralization of Cx43 and microtubule-associated proteins ↑	[94]
Rat	Pulmonary trunk banding with a diameter of 0.6 mm or 0.5 mm to induce compensated or decompensated HF, analysis after 7 w	Compensated and decompensated HF: Cx43 mRNA ↑	[95]

HF: heart failure; MCT: monocrotaline; PH: pulmonary hypertension; RA; right atrium; RVH: right ventricular hypertrophy; SERCA2a: sarcoplasmic reticulum calcium ATPase 2a; ↑: increased; ↓: decreased.

**Table 5:** Therapeutic approaches to improve RV function in PH involving connexins.

Species	Model	Result	Reference
Mouse	PAB or sham surgery, rhACE2 (1.8 mg/kg/d) or vehicle on post-op day 2, analysis 2 w after surgery	rhACE2: EF↑, RVH ↓; normalized the decreased Cx37 mRNA after PAB	[92]
Human	PAECs from PH patients treated with 1 µM MC1568 for 24 h	MC1568: normalized the decreased Cx37 and Cx40 expression	[67]
Rat	MCT (60 mg/kg) or saline, MC1568 (50 mg/kg) or saline daily	MC1568: RVSP ↓, RVH ↓	
Rat	MCT (60 mg/kg) or saline, after 3 w: intra-tracheal delivery of aerosolized AAV1.SERCA2a or no treatment, analysis after additional 3–4 w	AAV1.SERCA2a: reversal of electrophysiological remodeling; RV: normalized amounts of Cx43 mRNA and protein; normalized amounts of SERCA2a, of ion channels	[74]
Rat	MCT (60 mg/kg), MCT + nicorandil (5 mg/kg/d), MCT + colchicine (1 mg/kg/d), MCT + nicorandil + colchicine, analysis after 90 d	Compared to MCT alone: all groups showed RVSP ↓, RVH ↓, normalization of Cx43 protein	[84]
Rat	MCT (75 mg/kg), MCT + cilostazol (20 mg/kg/d, day 28–90) or saline, analysis after 90 d	Cilostazol: RVSP ↓, RVH ↓, Cx43 protein in RV and lung ↑	[86]
Rat	MCT (65 mg/kg), MCT + sildenafil (25 mg/kg/d, Day 21–90) or saline, analysis after 90 d	Sildenafil: RVSP ↓, RV weight ↓, Cx43 protein in RV ↑	[85]
Rat	MCT (60 mg/kg), MCT + bosentan (300 mg/kg/d, starting 2 w after MCT, or vehicle, analysis 5 w after MCT	Bosentan: RVSP ↓, RV/LV thickness ratio unchanged, prevention of Cx43 lateralization	[90]
Rat	MCT (60 mg/kg), MCT+ Carbenoxolone (20 mg/kg/d), analysis after 4 w	Carbenoxolone: RVH ↓; Cx43-expressing CD4^+^ and CD8^+^ cells in lungs of MCT-treated rats	[100]

EF: ejection fraction; HF: heart failure; MCT: monocrotaline; LV: left ventricle; PAEC: pulmonary arterial endothelial cells; PH: pulmonary hypertension; rhACE2: recombinant human angiotensin-converting enzyme 2; RVH: right ventricular hypertrophy; RVSP: right ventricular systolic pressure; SERCA2a: sarcoplasmic reticulum calcium ATPase 2a; ↑: increased; ↓: decreased.

## Data Availability

Full, uncut Western blot analysis is presented in Supplementary Materials Figure S2.

## Supplementary Materials

The following are available online at https://www.mdpi.com/1422-0067/22/3/987/s1, Figure S1: Purity of SSM and IFM isolated from mouse right and left ventricles. Figure S2: Expression of Cx43 in right and left ventricular tissue: unedited Western Blot images.
