# Supplementary material for: Importance of Cx43 for Right Ventricular Function
Source: Int J Mol Sci. 2021 Jan 20;22(3):987. doi: 10.3390/ijms22030987 (PMC7863922; doi:10.3390/ijms22030987)
Supplement: Supplementary file 1 [file ijms-22-00987-s001.pdf]

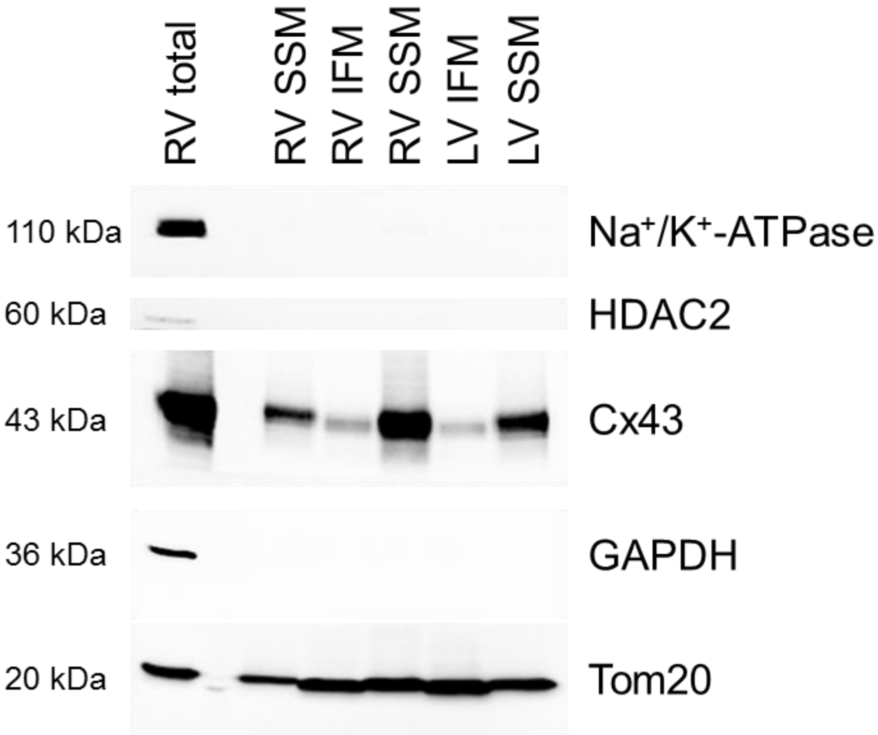

Figure S1. Purity of SSM and IFM isolated from mouse right and left ventricles. Western blot analysis demonstrates the purity of the subsarcolemmal (SSM) and interfibrillar mitochondria (IFM) isolated from mouse right (RV) and left (LV) ventricles by the absence of immunoreactivities for Na^+/K^+-ATPase, HDAC2 (histone deacetylase 2), and GAPDH (glyceraldehyde 3-phosphate dehydrogenase) as marker proteins for the sarcolemma, nucleus and cytosol, respectively. In addition, antibodies against Cx43 and the mitochondrial marker protein Tom20 (translocase of the outer membrane 20) were used. A RV total protein extract served as positive control.

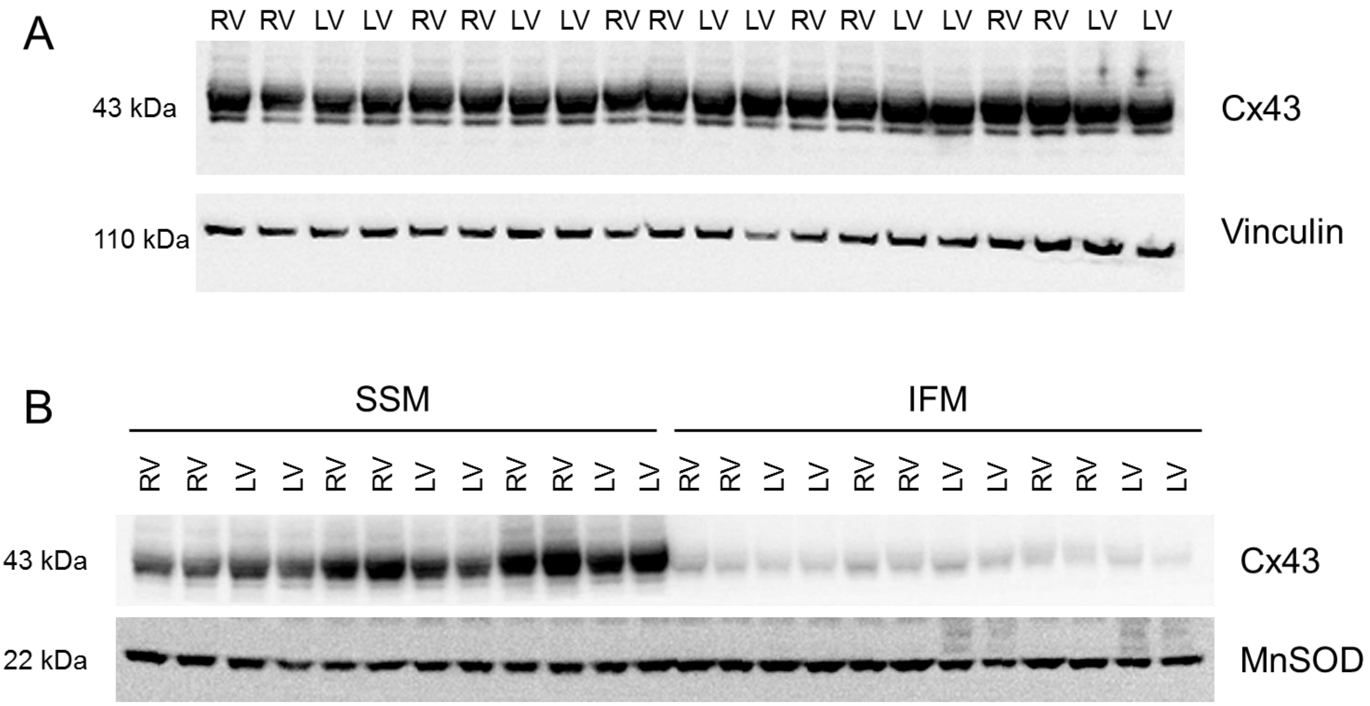

Figure S2. Expression of Cx43 in right and left ventricular tissue: unedited Western Blot images. **A:** Western blot analysis was performed on total mouse right (RV) and left (LV) ventricular proteins for Cx43 and Vinculin as a housekeeping protein. **B:** Western blot analysis was performed for Cx43 and MnSOD (manganese superoxide dismutase, mitochondrial marker protein) on subsarcolemmal (SSM) and interfibrillar (IFM) proteins isolated from mouse right (RV) and left (LV) ventricular tissue.
